# Transcription of the Human Microsomal Epoxide Hydrolase Gene (EPHX1) Is Regulated by PARP-1 and Histone H1.2. Association with Sodium-Dependent Bile Acid Transport

**DOI:** 10.1371/journal.pone.0125318

**Published:** 2015-05-20

**Authors:** Hui Peng, Qin-shi Zhu, Shuping Zhong, Daniel Levy

**Affiliations:** University of Southern California, Keck School of Medicine, Department of Biochemistry and Molecular Biology, Los Angeles, California, United States of America; Nihon University School of Medicine, JAPAN

## Abstract

Microsomal epoxide hydrolase (mEH) is a bifunctional protein that plays a central role in the metabolism of numerous xenobiotics as well as mediating the sodium-dependent transport of bile acids into hepatocytes. These compounds are involved in cholesterol homeostasis, lipid digestion, excretion of xenobiotics and the regulation of several nuclear receptors and signaling transduction pathways. Previous studies have demonstrated the critical role of GATA-4, a C/EBPα-NF/Y complex and an HNF-4α/CAR/RXR/PSF complex in the transcriptional regulation of the mEH gene (EPHX1). Studies also identified heterozygous mutations in human EPHX1 that resulted in a 95% decrease in mEH expression levels which was associated with a decrease in bile acid transport and severe hypercholanemia. In the present investigation we demonstrate that EPHX1 transcription is significantly inhibited by two heterozygous mutations observed in the Old Order Amish population that present numerous hypercholanemic subjects in the absence of liver damage suggesting a defect in bile acid transport into the hepatocyte. The identity of the regulatory proteins binding to these sites, established using biotinylated oligonucleotides in conjunction with mass spectrometry was shown to be poly(ADP-ribose)polymerase-1 (PARP-1) bound to the EPHX1 proximal promoter and a linker histone complex, H1.2/Aly, bound to a regulatory intron 1 site. These sites exhibited 71% homology and may represent potential nucleosome positioning domains. The high frequency of the H1.2 site polymorphism in the Amish population results in a potential genetic predisposition to hypercholanemia and in conjunction with our previous studies, further supports the critical role of mEH in mediating bile acid transport into hepatocytes.

## Introduction

Microsomal epoxide hydrolase (mEH) is a 48-kDa bifunctional protein that is expressed on the hepatocyte endoplasmic reticulum membrane in two distinct topological orientations [1] where the type I form plays a central role in the metabolism of numerous xenobiotics [2]. The type II form is targeted to the plasma membrane where it can mediate the sodium-dependent transport of bile acids [3–10] in parallel with the sodium-taurocholate cotransporting protein (Ntcp) [11]. The bile acids play a critical role in the digestion of dietary lipids, excretion of xenobiotics, and in the regulation of cholesterol homeostasis, nuclear receptors such as FXR and signal transduction such as the AKT and ERK1/2 pathways [12–14]. The regulation of bile acid transporter capacity/function is of critical importance in order to maintain the proper concentration and cellular distribution of the bile acids. Defects in bile salt transporters thus are involved in the etiology of numerous hepatobiliary disorders [15]. Previous studies from this laboratory have demonstrated that GATA-4 [16], a C/EBPα-NF/Y complex [17] and an HNF-4α/CAR/RXR/PSF complex [18] play critical roles in regulating the transcription of the mEH gene (EPHX1). Studies have also identified mutations in human EPHX1 that resulted in a 95% decrease in mEH expression that was associated with a significant decrease in bile acid uptake across the sinusoidal plasma membrane resulting in a 100-fold increase in serum bile salt levels (hypercholanemia) in the absence of liver damage [19]. In contrast, the Ntcp mRNA and protein expression levels in this subject were normal with no mutations in the amino acid sequence [20].

In order to further explore the role of mEH in sodium-dependent hepatocyte bile acid transport we investigated the occurrence of EPHX1 mutations in the Lancaster County Old Order Amish population that exhibit numerous cases of hypercholanemia [21] in the absence of hepatocellular injury suggesting a defect in bile acid uptake [22]. Linkage analysis identified several candidate genes [21] as well as a heterozygous region that contains the EPHX1 locus at 1q42.1 (L. Bull, personal communication). Sequencing and genotyping studies of EPHX1 have identified 2 functional mutations; one at a poly(ADP-ribose)polymerase-1 (PARP-1) binding site in the proximal promoter region (-17) and a second at a linker histone (H1.2) binding site in intron 1 (+2557), the latter mutation originally observed in our previous studies [19], which resulted in a significant decrease in EPHX1 promoter activity.

PARP-1 is a multifunctional nuclear protein that plays a critical role in numerous nuclear processes including gene regulation utilizing several mechanisms such as a) modulation of chromatin structure by binding to nucleosomes and b) functioning as a transcriptional regulator by binding to DNA through numerous related but non-identical sequences [23,24] resulting in the activation or repression of transcription. H1 linker histones play a critical role in regulating chromatin structure and gene expression through their interaction with nucleosomes where DNA sequence plays a significant role in the positioning, stability and activity of these structures where nucleosomes inhibit access of transcription factors to their DNA binding sites [25–27]. Histone H1 and PARP-1 also exhibit a reciprocal pattern of chromatin binding associated with actively transcribed genes where depletion of H1 by PARP-1 can result in increased transcription [28,29]. Linker histones may also regulate numerous processes through protein-protein interactions [30].

The results reported in this study demonstrate that EPHX1 transcription is regulated, in part, by PARP-1 and a linker histone H1.2/Aly complex. The similar DNA sequences at their respective binding sites in the proximal promoter and in intron 1 represent possible nucleosome positioning sites. The association between the high frequency of the intron 1 polymorphism resulting in a decrease in EPHX1 expression and the numerous cases of hypercholanemia in the Amish population further suggest that mEH plays a significant role in sodium-dependent bile acid uptake where the decreased expression of mEH is one factor in the etiology of hypercholanemia.

## Materials and Methods

### Sequencing of the EPHX1 gene

The promoter region (-784/-1), nine exons, intron 1, exon-intron boundaries and the 3’ untranslated regions were analyzed in a genomic DNA sample from a hypercholanemic subject (1d) [21] by sequencing fragments produced by PCR using the appropriate primers at the Microchemical Core Facility of the Norris Cancer Center at the Keck School of Medicine.

### Plasmids

To test the effect of mutations in the promoter region on EPHX1 promoter activity, the DNA from -784 to +25 containing the mutant or wild type DNA sequences were isolated as HingIII/BamHI fragments and cloned into the basic luciferase reporter construct pGL3 (Promega), where the linker sequence has been replaced by a new linker sequence 5’ AAGCTTCCCGGGCTCGAGATCTGAATTC-3’, which contains the restriction enzyme sites HindIII/SmaI/Xho1/BgiII/EcoRI. To differentiate the effect of each mutation, the DNA regions containing each mutation were exchanged between the *Hin*dIII(-784)/ScaI(-266) fragment which contained the -733 T>C mutation, the ScaI(-266)/XhoI(-103) fragment which contained the -179 T>C mutation and the XhoI(-103)/EcoR(+25) fragment, which contained the -17 T>C mutation. The core promoter fragments of the wild type and -17 mutant (-80 to +25) were also cloned into pGL3 vector. The -1797/+3458 construct containing the WT and C>G mutation as previously described [19] was inserted into the pGL3 vector. The expression plasmids for PARP-1 shRNA and Aly were obtained from Santa Cruz Biotechnology and for H1.2 and H1.2 shRNA from Dr. Woojin An (University of Southern California, Los Angeles, CA).

### Promoter activity assay

HepG2 cells were obtained from the American Type Culture Collection (ATCC) and grown at 37°C in Dulbecco’s modified Eagle’s medium supplemented with 10% fetal bovine serum. The wild type and mutant promoter constructs were transiently transfected into HepG2 cells using Lipofectamine 2000 (Invitrogen) according to manufacturer’s recommendations. Renilla luciferase reporter plasmid was cotransfected as an internal control to monitor transfection efficiency. The experiment was carried out in triplicate in 24-well plates. Forty-eight hours after transfection, the reporter luciferase activities were measured with the Dual-Glo Luciferase Assay System to measure both firefly and Renilla luciferase activities. The former was normalized to the latter as the EPHX1 promoter activity. Whenever expression vectors were used, empty vector was added in the control samples to keep total amount of added DNA constant (0.8 mg for each well). The assays were carried out in triplicate and repeated at least three times.

### Electrophoresis mobility shift assay (EMSA)

**Fig 1:**
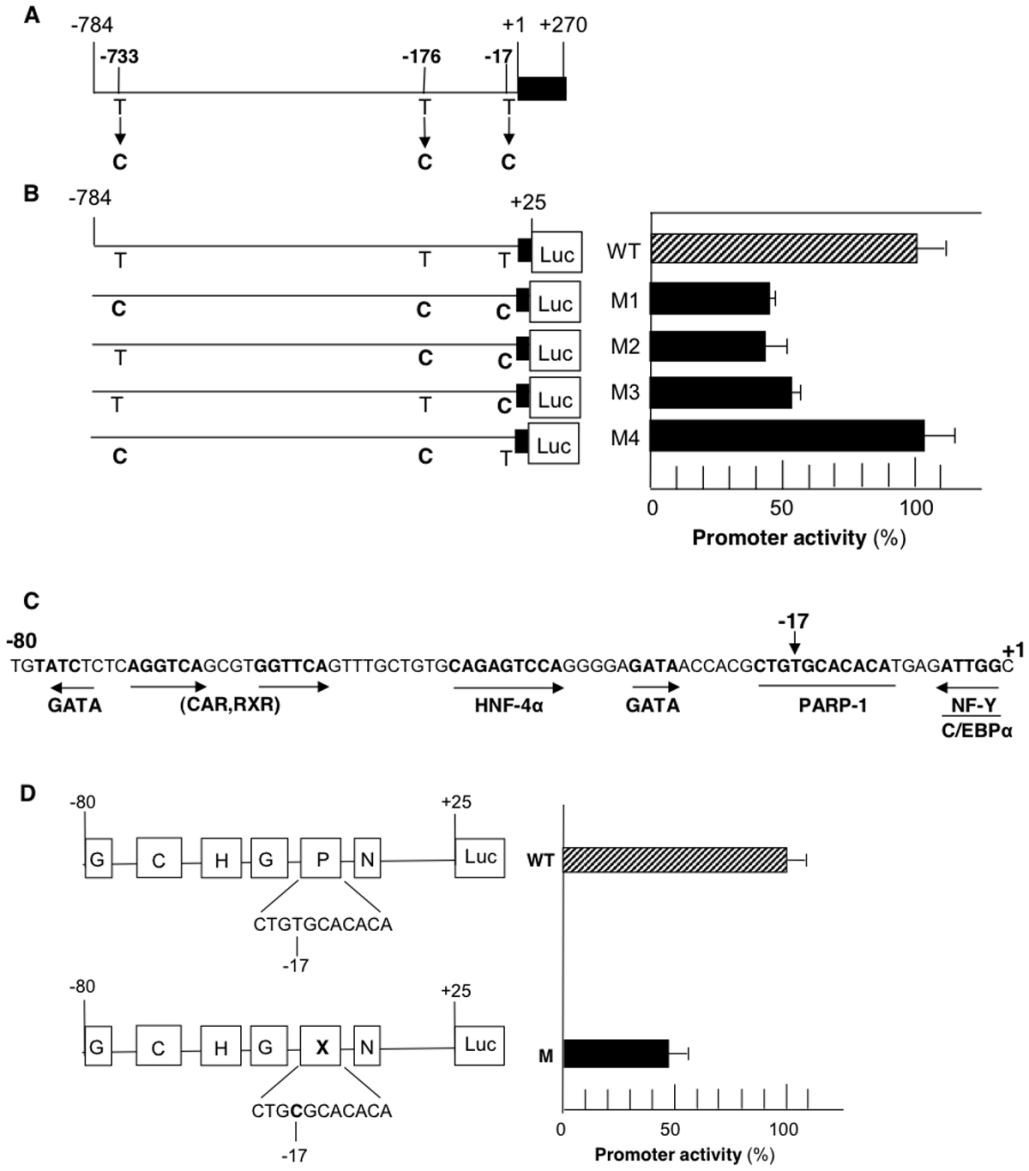
Analysis of EPHX1 transcriptional activity mediated by the proximal promoter region in HepG2 cells. A: Map of the EPHX1 promoter indicating the location of 3 naturally occurring T>C mutations observed in hypercholanemic subject 1d [21]. B: The effect of the T>C mutations on EPHX1 promoter activity. WT and M forms of the -80/+25 EPHX1 promoter region were inserted into the pGL3 expression vector containing the luciferase reporter gene (LUC). Values are the mean +/- S.D. of independent experiments (n = 3–4) performed in triplicate. *P*<0.01 for all studies reported. C: Nucleotide sequence of the EPHX1 proximal promoter (-80/+1) indicating the location of the critical -17 T>C substitution within the PARP-1 binding site as well as neighboring transcription factor binding sites previously described [16–18]. D: The effect of the -17 mutation on EPHX1 promoter activity driven by the -80/+25 promoter.

Nuclear proteins were extracted from cultured HepG2 cells as previously described [16]. Sense and antisense oligonucleotides were synthesized, annealed and radiolabeled with ^32^P-dCTP in fill-in reactions with the Klenow large fragment of DNA polymerase I. The sequence of the sense strand is given below. The probe for the -17 PARP-1 binding site, corresponded to -28/-4 bp of the EPHX1 gene and its mutants are given in Fig 1C. The PARP-1 binding probe (Px) of unrelated sequence [24] was 5’-ATCTATTGATATAAATGTATCTATTTATTGATTCTAGCTG-3’. In super-shift assays, antibodies against PARP-1 and Aly (Santa Cruz Biotechnology) and H1.2 (Abcam) were incubated with the reaction mixture on ice for 2 h before loading on the gel. The EMSA was carried out as previously described [16] and the protein-DNA bands were visualized by autoradiography.

### Identification of PARP-1 and H1.2

20 μl of BioMag beads (Sigma) were washed three times with PBS, and suspended in 20 μl of binding buffer (20 mMTris-HCl, pH7.8, 1 mM Mg_2_Cl, 50 mM NaCl, 0.5 mM EDTA, 0.5 mM DTT and 10% glycerol) after removing PBS by magnetically absorbing the beads on the tube wall. The beads were incubated at room temperature (RT) for 20 min with 2 pmol of a biotinylated oligonucleotide (-28/-4) containing the intact PARP-1 binding site (a) or a biotinylated oligonucleotide (+2508/2607) containing the H1.2 binding site (b). The beads were washed with 1x binding buffer and then suspended in 500 μl H_2_O, 200 μl 5x binding buffer, 50 μl fat-free BSA (100mg/ml) for 20 min at RT. The beads were mixed with 50 μl poly dI.dC (2.5μg/μl) and 200 μl HepG2 nuclear proteins (600 μg) and 5 μl 1M DTT and incubated at RT for 90 min. The beads were washed and eluted to collect the oligonucleotide-binding proteins. This mixture was run on SDS-PAGE, silver stained and a band containing protein (113 kDa) (a) or 2 bands (29–32 kDa) (b) were excised and released from the gel by trypsinization and analyzed by mass spectrometry on a Bruker Autoflex III MALDI TOF/TOF instrument.

### Co-immunoprecipitation assay and Western blotting

HepG2 nuclear extracts (300 μg) were incubated with 15 μl of protein A/G-agarose beads and 8 μg anti-H1.2 or Aly antibodies as previously described (17). Pre-immune IgG was used as a negative control. Eluted proteins were separated by SDS-PAGE and electrophoretically transferred to a polyvinylidine difluoride membrane (Millipore). Proteins were detected with the indicated antibodies in a dilution of 1:100 to 1:500 with a Western Breeze Chromogenic Western Blot Immunodetection Kit (Invitrogen). Subconfluent HepG2 cells were harvested by trypsinization and lysed with universal lysis/immunoprecipitation buffer (50 mM Tris-HCl, pH 7.5, 150 mM NaCl, 2 mM EDTA, 2 mM EGTA, 25 mM NaF, 25 mM β-glycerophosphate, 0.1 mM sodium orthovanadate, 0.1 mM PMSF, 5 μg of leupeptin/ml, 0.5%(v/v) Triton X-100, 0.5% (v/v) Nonidet (P-40). Protein concentration was determined by the Bradford method (Bio-Rad). Western blotting was performed as described above.

### Chromatin immunoprecipitation (ChIP)

HepG2 cells were grown in Dulbecco’s Modified Eagle’s Medium supplemented with 10% fetal bovine serum to 80% confluency in a 15 cm plate and chromatin was prepared using ChIP-IT Express Kit (Active Motif) according to the manufacturer’s recommendation. All the buffers and reagents described below were provided by the manufacturer. Briefly, cells were fixed in 20 ml minimal cell culture medium containing 1% formaldehyde for 10 min and fixation was stopped by Glycine-Stop Fix solution. Cells were scraped and collected by centrifugation and lysed in lysis buffer for 30 min on ice. Cells were then dounce homogenized and nuclei were collected by centrifugation. Nuclei were resuspended in digestion buffer and chromatin was optimally sheared to yield bands between 100–200 bp as assessed by agarose gel analysis, by adding Enzymatic Shearing Cocktail and incubating for 8–10 min at 37°C. Sheared chromatin was collected by centrifugation at 15,000 rpm for 10 min at 4°C. Immunoprecipitation was performed using 7 μg of sheared chromatin, 3 μg of specific antibodies or pre-immune IgG (Santa Cruz Biotechnology), and 25 μl protein G magnetic beads (Active Motif). After a 4 h incubation on a rotator at 4°C, beads were washed and chromatin was isolated by elution buffer followed by reverse cross linking at 95°C for 15 min and proteinase K treatment for 1h at 37°C. PCR was performed with the following primers specific for a) EPHX1–297/+25 region (5’ primer: 5’-CTGTAAGGCACCCATCCTTGAGCC-3’; 3’primer: 5’-CAATTGCACAGTCCTGCCAAGTCAG-3’), b) EPHX1 +2462/+2641 region (5’ primer; 5’-TGTGAATATCCTAGAAACCACTGG-3’; 3’primer: 5’-GTAATGGGGAGGCCCCAAGTGCTG-3’) c) EPHX1–466/-301 region (5’ primer: 5’-CAGCGGGGTTGGAAACCCAC-3’; 3’ primer: 5’-CTGATCTCAGATTACAAATAG-3’). PCR products were resolved on a 1% agarose gel and visualized with ethidium bromide.

### Ethics Statement

Coded genomic DNA samples were obtained from Dr. Gerald Salen, New Jersey Medical School, N.J. who had obtained IRB approval for his studies at the above institution. This research projects was also approved by the IRB (#982059) at the University of Southern California, Keck School of Medicine.

## Results

### Sequencing of EPHX1 from an Amish hypercholanemic subject

Sequencing of the EPHX1 5’ region (-784/-1), 9 exons, intron 1, exon-intron boundaries and the 3’ untranslated region from a previously reported subject (1d) [21] that does not express the intron polymorphism previously reported [19] resulted in the identification of 3 heterozygous mutations in the proximal promoter at -17, -176 and -733 as shown in Fig 1A.

### Effects of T>C mutations at -17, -176 and -733 on EPHX promoter activity

The WT and 4 constructs containing the different permutations of the observed mutations (-784/+25) were cloned into the luciferase reporter plasmid pGL3 and were transfected into HepG2 cells and luciferase activity measured after 48 h. Only the constructs containing the -17 T>C substitution resulted in a 50–60% reduction in promoter activity (M1-3) while T>C substitutions at -733 and -176 had no effect on activity (M4) (Fig 1B). Previous studies have demonstrated that the -80/+25 proximal promoter for EPHX1 contains binding sites for numerous transcription factors (GATA, CAR/RXR, HNF-4α, NF-Y) as shown in Fig 1C. The functional role of the T>C substitution was confirmed by incorporating the WT and -17 mutant into the EPHX1 reporter construct (-80/+25) pGL3) followed by transient transfection into HepG2 cells as previously reported resulting in a 65% reduction in promoter activity (Fig 1D). The -17 mutation was only found in one other subject (1b) [21] from the Amish population.

### Identification of a transcription factor binding to the -17 region in the EPHX1 proximal promoter

DNA affinity chromatography using a biotinylated oligonucleotide (-28/-4) in conjunction with streptavidin-coated magnetic beads resulted in the isolation by SDS-PAGE of a 113-kDa protein. The band, visualized by silver staining, was cut from the gel and sequenced by mass spectrometry and identified as poly(ADP-ribose) polymerase.

### The interaction of PARP-1 with EPHX1

**Fig 2:**
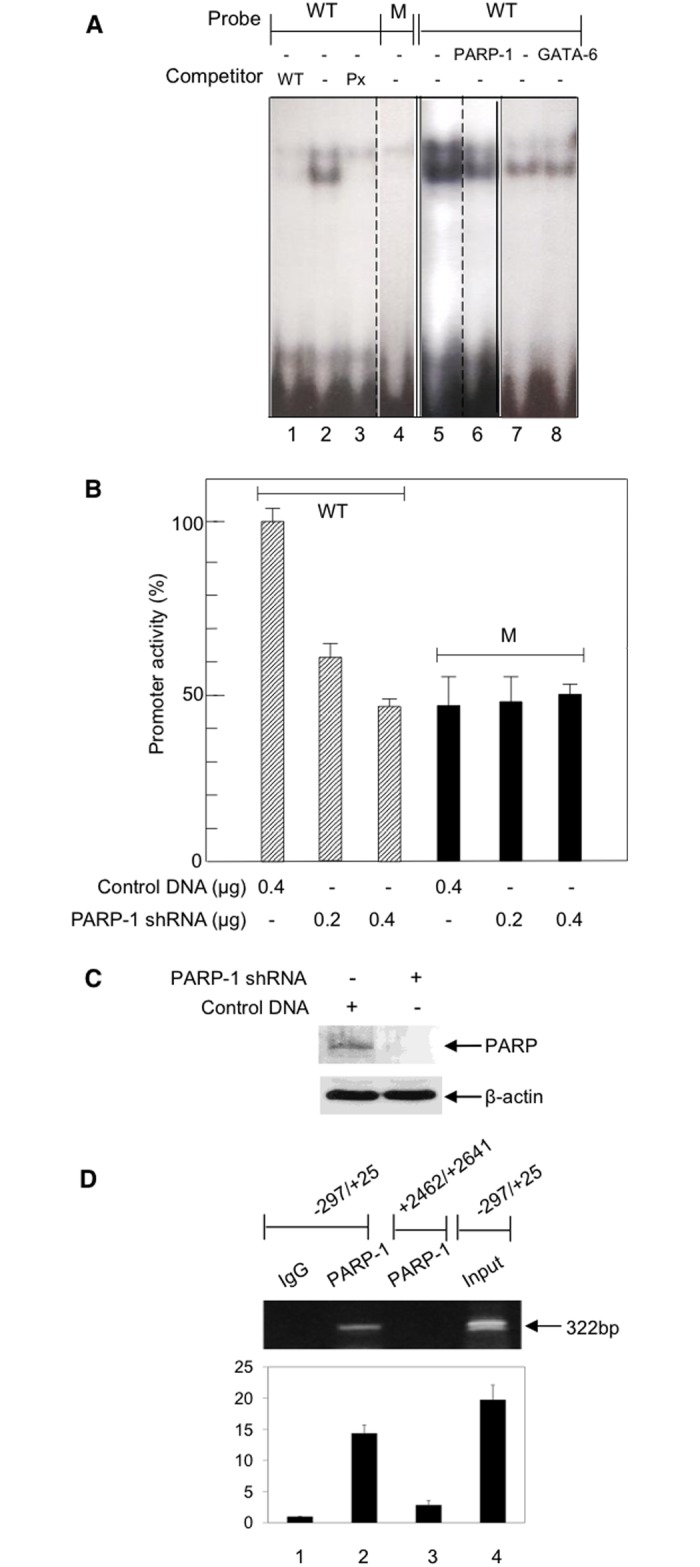
Characterization of the interaction of PARP-1 with the -20/-10 EPHX1 promoter region. A. EMSA analysis was performed with a HepG2 nuclear protein extract and ^32^P-labeled oligonucleotides (-28/-4) containing the WT PARP-1 sequence in the absence and presence of a 50-fold excess of unlabeled oligonucleotide WT competitor (lane 2,1), and in the presence of a 50-fold excess of an oligonucleotide (Px) of unrelated sequence that can also binds PARP-1 (lane 3). EMSA was performed with the labeled -17 mutated oligonucleotide (lane 4). Nuclear extracts were preincubated with a PARP-1 antibody (lane 5,6) or a GATA-6 antibody (lane 7,8) prior to EMSA analysis. B. The effect of PARP-1 shRNA on the promoter activity of the EPHX1 -8-/+25 construct. Values are the mean +/- S.D. of independent experiments (n = 3–5) performed in triplicate. C. Western blot of PARP-1 levels following treatment with 0.4 μg PARP-1 shRNA. D. The interaction of PARP-1 with the EPHX1 proximal promoter as assessed by ChIP analysis. Crosslinked protein-DNA complexes were incubated with anti-PARP-1 antibody, isolated by protein A-Sepharose beads and DNA corresponding to the EPHX1–297/+25 and EPHX1 +2462/+2641 which does not contain the PARP-1 binding site were analyzed by PCR. The band observed for PARP-1 in the EPHX1 promoter region (lane 2). Immunoprecipatation with IgG (lane 1). PCR analysis with primers to the +2462/+2641 region (lane 3). Gels were scanned and quantitated using UN-SCAN-IT gel automated digitizing system. Values are the result of 3 independent experiments.

EMSA analysis was performed using extracts from HepG2 cells and probes for the WT and -17M sequence to establish that PARP-1 binds to the proximal EPHX1 promoter region. As shown in Fig 2A, a probe containing the -28/-4 bp sequence formed a DNA-protein complex (lane 2) which could be inhibited by a 50-fold excess of unlabeled probe (WT) (lane 1) while the use of the probe containing the -17 mutation failed to form a complex with PARP-1 (lane 4). The formation of the complex could also be inhibited by a probe from a previously identified PARP-1 binding site which had a unrelated sequence [lane 3] suggesting that PARP-1 binding was dependent on the topology of the oligonucleotide [24] formed by more than one sequence. Serial mutational analysis determined the DNA sequence essential for binding to be from -20/-10 (5’-CTGTGCACA-3’) (data not shown). To identify the components of this complex, EMSA analysis was carried out in the presence of a PARP-1 specific antibody which resulted in a large decrease in complex formation (lane 5,6). In contrast, no effect on complex formation was observed when a GATA-6 specific antibody was used (lane 7,8). The functional role of PARP-1 was established by treating HepG2 cells containing the WT or -17 T>C variant EPHX1 promoter plasmids (-80/+25) with PARP-1 shRNA as described in the Materials and Methods. A significant concentration dependent decrease in promoter activity was observed only when the WT plasmid was utilized (Fig 2B). The shRNA dependent loss of promoter activity was associated with the decreased expression of PARP-1 protein (Fig 2C). ChIP analysis was then utilized to establish that PARP-1 interacted with the domain (-20/-10) containing the PARP-1 binding motifs on the endogenous EPHX1 promoter. Cross-linked chromatin from HepG2 cells was immunoprecipitated with a PARP-1 specific antibody and the presence of the EPHX1 promoter fragment established by PCR using primers amplifying the region from -297/+25. A negative control was obtained by PCR amplification using primers (+2462/+2641) that bind to a downstream region that does not contain the PARP-1 binding motif. As shown in Fig 2D a PARP-1 antibody immunoprecipitated a fragment located in the EPHX1 proximal promoter (lane 2). No band was observed when the downstream region was amplified (lane 3) or when IgG was used (lane 1). These results establish that PARP-1 forms a complex that binds to the EPHX1 promoter *in vivo*.

### Identification of a protein binding to the EPHX1 intron 1 +2557 mutation site region

**Fig 3:**
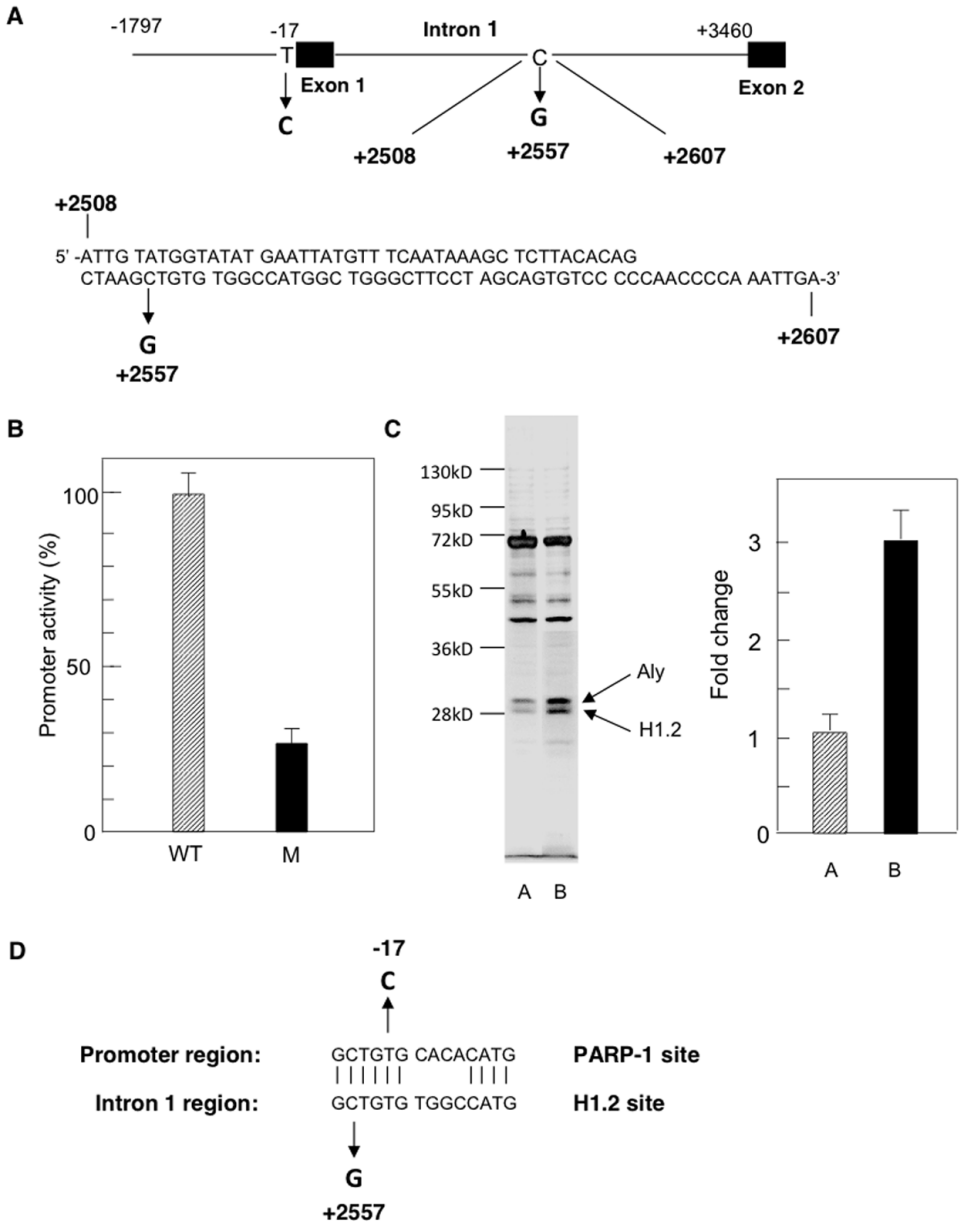
Characterization of the interaction of H1.2 with the EPHX1 intron 1 region. A. Map showing the EPHX1 promoter indicating the -17 T>C mutation and the location of the intron 1 polymorphism at +2557 and the sequence of the oligonucleotide used to isolate the H1.2/Aly complex. B. The effect of the C>G at +2557 on EPHX1 transcription using the -1797/+3460 fragment in the pGL3 vector containing the luciferase reporter gene. Values are the mean +/-S.D. of independent experiments (n = 3–5) performed in triplicate C. DNA affinity chromatography in conjunction with streptavidin-coated magnetic beads using the WT (lane 1) and C>G mutated (lane 2) 100 bp oligonucleotides (+2508/+2607) analyzed by SDS-PAGE and quantitated using the UN-SCAN-IT gel automated digitizing system. The bands were cut from the gel and identified by mass spectrometry. D. Comparison of the sequences in the PARP-1 and H1.2 binding sites.

Previous studies have demonstrated that a C>G substitution in EPHX1 intron 1 at +2557 resulted in a 75% decrease in EPHX1 promoter activity [19]. Transfection of the WT and +2557C>G mutant constructs (-1797/+3460) in pGL3 into HepG2 cells again showed a 72% decrease in promoter activity (Fig 3B). Database analysis of the sequence in the +2557 region failed to identify any known transcription factor binding site. Because of the palindromic nature of this site (Fig 3A) (+2545/2561) a 100 bp oligonucleotide was utilized in order to decrease the formation of a single-stranded hairpin loop that may have occurred using the shorter 29 bp oligonucleotide (+2540/+2569) [19]. As described above for PARP-1, DNA affinity chromatography using a WT biotinylated oligonucleotide (+2508/2607) in conjunction with streptavidin-coated magnetic beads resulted in the visualization by silver staining of SDS-PAGE of 2 bands (28–31 kD) (Fig 3C, lane A). The bands were cut from the gel, sequenced by mass spectrometry and identified as a) linker histone H1.2 and b) Aly. When the 100 bp oligonucleotide containing the C>G substitution was used, a 3-fold increase in the intensity of the H1.2 and Aly bands was observed (Fig 3C, lane B). A comparison of the PARP-1 (Fig 1C) and the H1.2 (Fig 3A) binding site sequences indicated a 71% homology (Fig 3D) which is consistent with the previously reported reciprocal binding of these proteins [29].

### The interaction of H1.2 with EPHX1

**Fig 4:**
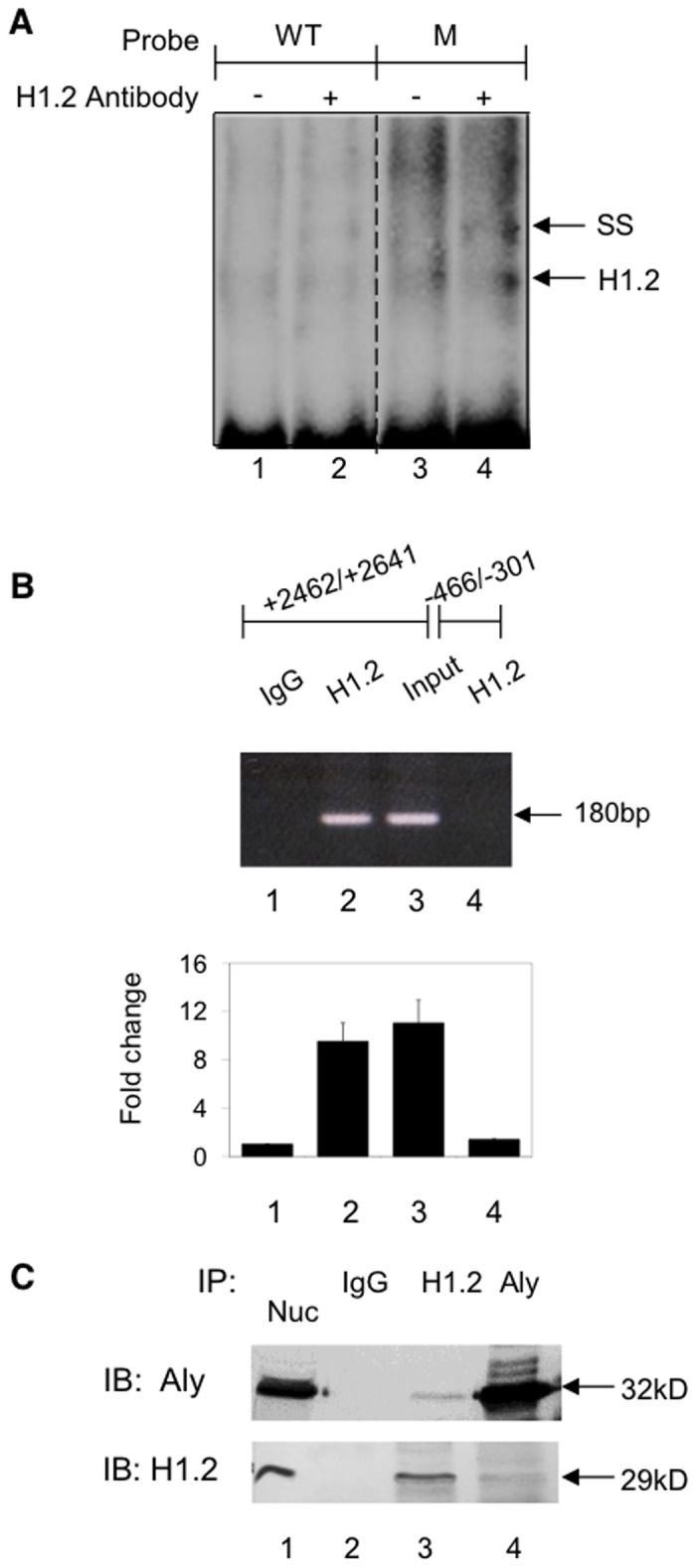
Characterizing the interaction of H1.2 and Aly with the EPHX1 intron 1 WT and +2557 C>G mutated region. A. EMSA analysis was performed with a HepG2 nuclear protein extract and ^32^P-labeled oligonucleotides (Fig 3A) containing the WT (lane 1) and mutated sequence (lane 3). Nuclear extracts were preincubated with a H1.2 antibody (lane 2,4) prior to EMSA analysis. SS indicates the supershifted band. B. The interaction of H1.2 with the EPHX1 intron 1 polymorophic region assessed by ChIP analysis. Crosslinked protein-DNA complex was incubated with anti-H1.2 antibody, isolated by protein A-Sepharose beads and DNA corresponding to EPHX1 +2462/+2641 and EPHX1–466/-301 as a control region were analyzed by PCR. Band observed for H1.2 in intron 1 (lane 2). Immunoprecipitation with IgG (lane 1). PCR analysis using primers to the control 466/-301 domain (lane 4). Results analyzed as in Fig 2D. C. HepG2 cell nuclear extracts were immunoprecipitated (IP) with anti-H1.2 antibody (lane 3) and anti-Aly antibody (lane 4) or non-specific IgG (lanes 2) and analyzed by SDS-PAGE followed by immunoblot (IB) analysis with anti-Aly and anti-H1.2. Nuclear extracts immunoblotted with anti-Aly and anti H1.2 antibodies (lane 1).

To further establish that H1.2 binds to the intron region containing the +2557 mutation, EMSA analysis was performed using the WT and the C>G substituted 100 bp oligonucleotide probes described above. As shown in Fig 4A the WT probe formed a DNA-protein complex band (lane 1). When this analysis was carried out in the presence of a H1.2 specific antibody a supershifted band was now observed (lane 2). Using the M probe resulted in the formation of a more intense band (lane 3) as expected from the SDS-PAGE analysis shown in Fig 3C. Incubation with the H1.2 antibody also resulted in a supershifted band (lane 4). ChIP analysis was also utilized to establish that H1.2 interacted with the +2557 intron 1 domain on the endogenous EPHX1 promoter. Cross-linked chromatin from HepG2 cells was immunoprecipitated with a H1.2 specific antibody and the presence of the EPHX1 fragment established by PCR using primers amplifying the region from +2462/+2641. A negative control was obtained by PCR amplification using primers (-466/-301) that bind to a region that does not contain the H1.2 binding motif. As shown in Fig 4B, the H1.2 antibody immunoprecipitated a fragment located in intron 1 (lane 2). No band was observed when the upstream region was amplified (lane 4) or when IgG was used (lane 1).

### The interaction of H1.2 with Aly

The interaction of DNA bound H1.2 with Aly was investigated by immunoprecipitation of HepG2 nuclear extracts with anti-H1.2 and anti-Aly antibodies. The precipitated proteins were separated by SDS-PAGE and immunoblotted with the respective antibodies. As shown in Fig 4C, the addition of an anti-H1.2 antibody to nuclear extracts resulted in the immunoprecipitation of H1.2 as well as Aly (lane 3) while the addition of an anti-Aly antibody resulted again in the precipitation of H1.2 and Aly (lane 4). Protein bands were not detected when immunoprecipitation was carried out in the presence of non-specific IgG/protein A/G-agarose (lane 2). These results demonstrated that ALY interacts with DNA bound H1.2.

### The effect of H1.2 and ALY on EPHX1 promoter activity

**Fig 5:**
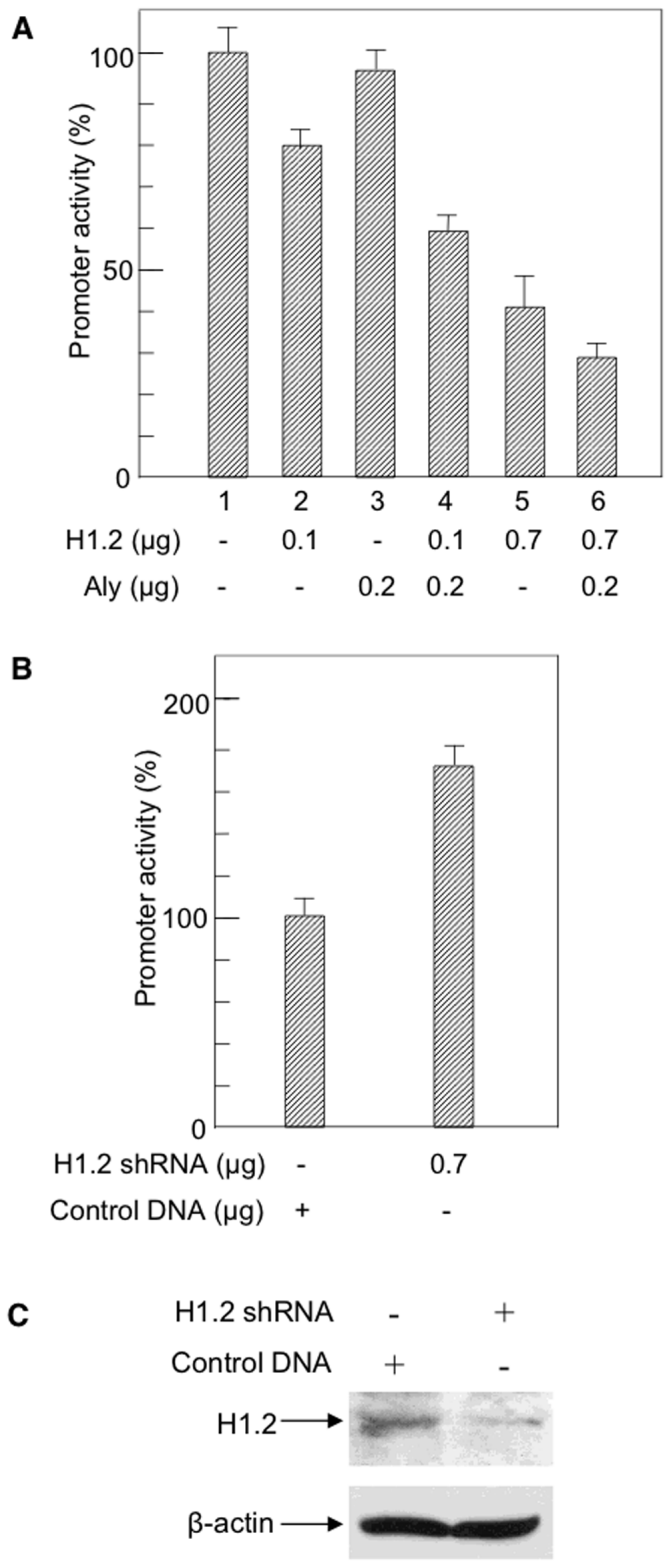
The effect of H1.2 and Aly on EPHX1 promoter activity. A. EPHX1 promoter activity using the -1797/+3460 construct (Fig 3) was measured in HepG2 cells expressing H1.2 (lane 2,5), ALY (lane 3) and H1.2 and ALY (lanes 4,6). B. The effect of H1.2 shRNA on EPHX1 promoter activity. Values are the mean +/- S.D. of independent experiments (n = 3–4) performed in triplicate. C. Western blot of H1.2 levels following treatment with 0.7 μg of H1.2 shRNA.

The functional role of H1.2 and Aly was further established by cotransfecting the H1.2 and/or Aly expression plasmids into HepG2 cells containing the EPHX1 WT plasmid (-1797/+3460). As shown in Fig 5A, expression of H1.2 resulted in a concentration dependent decrease in EPHX1 promoter activity (lane 2,5) while expression of Aly alone had no significant effect (lane 3). However, ALY did increase the inhibition of EPHX1 activity when cotransfected with H1.2 (lane 4,6). Treatment of HepG2 cells containing the EPHX1 expression plasmid with H1.2 shRNA resulted in a significant increase in EPHX1 promoter activity (Fig 5B) as well as corresponding decrease in H1.2 protein (Fig 5C). These results confirm that H1.2 binding to the EPHX1 intron 1 region around +2556 results in the inhibition of EPHX1 promoter activity.

### Genotype analysis of the EPHX1 intron 1 polymorphism in the Lancaster County Old Order Amish population

The association between the expression levels of mEH and hypercholanemia in the absence of hepatocellular injury indicating aberrant bile acid uptake was studied to further support the role of mEH in sodium-dependent bile acid transport. Genomic DNA samples from the Amish population [21] which presents numerous hypercholanemic subjects were subjected to genotype analysis for the EPHX1 intron 1 polymorphism as previously described for populations of differing ethnic backgrounds [31]. The frequency of this heterozygous C>G polymorphism at +2556 was significantly higher in the Amish population (62%) than observed in other ethnic groups (4–19%) suggesting a genetic predisposition for reduced mEH expression and bile acid uptake contributing to the observed hypercholanemia.

## Discussion

The expression of mEH and its subsequent functional role in xenobiotic metabolism and sodium-dependent bile acid transport is determined by several transcription factors binding to the proximal promoter. Previous studies have demonstrated that the transcriptional regulation of EPHX1 is mediated, in part, by GATA-4 [16], and complexes composed of C/EBPα-NF/Y [17] and HNF-4α/CAR/RXR/PSF [18]. Based on polymorphisms in the Amish population, this study has establish that EPHX1 expression is also significantly inhibited by a mutation at -17 within a sequence that did not contain a known transcription factor binding site (Fig 1C). DNA affinity chromatography, mass spectrometry, EMSA (Fig 2A), plasmid transfection (Fig 2B) and ChIP analyses (Fig 2D) confirmed the regulatory role of PARP-1 at this site. Previous studies have demonstrated that PARP-1 interacts with GATA [29] and C/EBPα [32] which are found to bind to adjacent domains in the EPHX1 promoter (Fig 1C), and thus may serve to further stabilize this interaction. The binding of PARP-1 to this site may exert its effect on transcription by modifying chromatin structure, by competing with histone H1 to maintain a transcriptionally competent environment or by acting as a transcription factor interacting with RNA polymerase II.

EPHX1 expression was also inhibited by a mutation at +2557 in intron 1 (Fig 3B), a mutation that had been previously been reported to be found in a non-Amish hypercholanemic subject [19]. DNA affinity chromatography (Fig 3C) and mass spectrometry identified linker histone H1.2 binding to this domain while EMSA (Fig 4A), plasmid transfection (Fig 5A, 5B, and 5C) and ChIP analysis (Fig 4B) confirmed its regulatory role. The isolation of H1.2 using the 100 bp oligonucleotide (Fig 3A) resulted in the co-isolation of Aly whose interaction with H1.2 was established by co-immunoprecipitation and immnoblotting (Fig 4C). The functional efficacy of this interaction was demonstrated where Aly increased the inhibitory properties of H1.2 (Fig 5A). Aly has been shown to be a component of the mRNA export machinery [33] as well as modulating gene expression by acting as a coactivator of RUNX1, cMyb [34] and E2F2 transcription factors [35]. In the latter study the interaction of ALY with H1.2 was also observed. The -17 mutation resulted in the loss of PARP-1 binding (Fig 2A) and a decrease in EPHX1 promoter activity (Fig 1D). In contrast, the +2557 mutation resulted in a significant increase in binding (Figs 3C and 4A) of the inhibitory H1.2/Aly complex (Fig 5A) resulting in the observed inhibition (Fig 3B) using the -1797/+3460 construct (Fig 3A) further suggesting that H1.2 may act as a repressor by affecting nucleosome positioning and chromatin structure [25–27].

The role of linker histones and the mechanism of nucleosome positioning is essential for the understanding of gene regulation. Factors influencing this process such as DNA sequence preference, histone variants and post-translational modifications have been extensively investigated [25,26]. Studies have demonstrated a large range of affinities for different DNA sequences which is thought to be caused by sequence-dependent mechanics of wrapped DNA. A change in 2 bp in a 200-bp system has been shown to change affinities by approximately 500-fold [27]. This sensitivity to sequence is reflected in the 1-bp change observed at +2557 in EPHX1 resulting in a significant increase in DNA affinity with a concomitant decrease in EPHX1 promoter activity. Part of the sequence in the region surrounding the +2557 polymorphism is quite similar to those previously reported as putative linker histone binding sites [36,37]. In addition, there is a 71% homology between the H1.2 and PARP-1 binding sites (Fig 3D) which is consistent with the report that PARP-1 and H1 can compete for DNA binding sites [28,29]. The observed differential binding of H1.2 to the WT and mutated +2508/2607 EPHX1 fragment therefore may serve as a model for its binding when it is a component of the octomeric histone cluster of the nucleosome. In this regard studies have demonstrated that H1 is able to bind to DNA in the absence of nucleosomes resulting in DNA compaction but to a lesser degree [38].

The analysis of genomic DNA samples from the Amish population for the intron 1 G>C mutation at +2557 that affects H1.2 binding, first observed in an independent hypercholanemic patient [19], indicated that a large percentage of this population carried this heterozygous polymorphism (62%) compared to multiple ethnic populations (4–19%) [31] indicating a potential genetic predisposition for reduced hepatic mEH expression and bile acid uptake capacity. As initially observed, the presence of the heterozygous mutation alone was not sufficient to cause hypercholanemia but required additional mutations such as in an HNF-3α binding site as previously reported [19]. In addition to mutations in EPHX1, linkage analysis identified 2 additional candidate genes, namely tight junction protein 2 (TJP2) and bile acid coenzyme A:amino acid N-acyltransferase (BAAT) [21] that have been associated with hypercholanemia by suggested mechanisms involving back diffusion or leakage of bile acids into the sinusoidal compartment, however their quantitative contribution to the etiology of this syndrome has not been resolved. For example: a) several non-affected siblings expressed the homozygous TJP2 mutation; b) a non-affected sibling (7f) expressed the homozygous BAAT mutation (L.Bull, personal communication); c) several affected siblings that are only homozygous for the BAAT mutation (9e-g) or only the TJP2 mutation (2c,5c) also contained the H1.2 mutation; d) one patient (12c) did not express the TJP2, BAAT or H1.2 mutations. In addition, no mutations were found in the Ntcp gene in this subject suggesting the possible existence of other factor(s) involved in this pathology. These results suggest a complex etiology for this syndrome where more than one of the above-described mutations is necessary to cause a sufficient decrease in net bile salt transport from the sinusoidal to the biliary compartment by one or more of these mechanisms resulting in hypercholanemia.

The sodium-dependent uptake of bile acids by hepatocytes mediated by Ntcp is well documented [11], however the relative physiologically pertinent contribution of mEH and Ntcp to this process in rodents and humans remains unresolved for reasons outlined below and has thus been the source of considerable controversy. Despite our publications [1,3–10,19] numerous papers over a period of 20 years continue to reference a study [39] that reported the inability to observe the expression of bile acid transport in a stably transfected cell line (BHK21) that expressed high levels of mEH. These studies, however, were unable to demonstrate that mEH was targeted to the cell surface and therefore could not draw any conclusions concerning the transport capacity of mEH. In contrast, our studies with stably transfected MDCK cells expressing mEH, clearly demonstrated targeting to the plasma membrane [10] and sodium-dependent bile acid transport [9]. In addition, the bile acid transport capacity of hepatocytes in culture showed a time dependent decrease over 72 h that paralleled the loss of mEH expression on the cell surface [10]. mEH was also not targeted to the plasma membrane of HepG2 cells. [10]. We have previously elucidated the effect of the topological orientation of mEH in the endoplasmic reticulum on the targeting of mEH to the hepatocyte plasma membrane [1]. The additional factor(s) affecting mEH targeting in cultured hepatocytes and in HepG2 cells [10] remain to be characterized.

In regard to Ntcp, initial studies using *Xenopus laevis* oocytes showed a 95% reduction of sodium-dependent taurocholate uptake following inhibition of Ntcp expression by antisense nucleotides [40] suggesting that Ntcp is the main bile acid transport mediator, however these studies failed to establish if mEH was expressed and targeted to the plasma membrane of this system. In addition the oligonucleotide used was not specific for Ntcp [41]. Studies in the *fch/fch* mouse, which is a model of erthropoietic protoporphyria, demonstrated that Ntcp and the sodium-independent bile acid transporter, OATP-1 were undetectable, while mEH levels remained unchanged (unpublished observation). Studies demonstrated that a majority of intravenously administered [^3^H]taurocholate was still observed in the liver and bile compartments after 30 min [42]. In mice treated with the peroxisome proliferator-activated receptor α (PPARα) activator, ciprofibrate, protein levels of the canalicular bile acid transporter (BSEP) as well as Ntcp and OATP-1 were undetectable or drastically reduced. Despite the loss of these transporters the plasma bile acid concentration and biliary bile acid secretion rates were unaffected [43]. In studies utilizing a different PPARα activator, Wyeth 14643 (pirinixic acid), the expression of mEH was increased 3-fold in rats and mice [44,45]. Studies using the BSEP knockout mouse (BSEP^-/-^) also reported a 2–3 fold increase in mEH expression [46]. Studies have also demonstrated that the uptake of fluorescent bile acids in primary rat hepatocytes does not correlate with the expression of Ntcp [47]. There is also a discordance between bile acid transport function and the expression of Ntcp. Ontogenic expression in vesicles and hepatocytes indicate that bile acid uptake first appears on fetal day 20 and at 14 days is 50% of that observed in adult liver, however Ntcp is not expressed in its mature glycosylated form for 4 weeks [48–51]. In contrast, mEH expression parallels bile acid transport capacity during development [7]. This observation is in concert with studies which showed that an antibody against a 48 kDa protein which is expressed in parallel with bile acid transport function inhibited bile acid transport [52]. This protein has the same molecular weight and isoelectric point (9.0) as mEH. Reconstitution studies indicated that this protein was required for bile acid transport function [52] as we previously reported for mEH [6].

A recent study [53] reports that a mutation in the human Ntcp gene results in a protein variant that is no longer targeted to the plasma membrane. The subject exhibits elevated bile acids in the serum in the absence of liver damage which is postulated to result from the loss of Ntcp mediated bile acid uptake. While this is a reasonable conclusion, the study does not characterize the expression levels of other possible bile acid transporters or reference the studies of a hypercholanemic subject with a 100-fold increase in serum bile acid levels without liver damage that exhibits normal Ntcp expression levels and protein sequence but with a 95% decrease in mEH expression [19,20] or the ciprofibrate studies in mice [43] that demonstrated normal bile acid serum levels in the absence of Ntcp and OATP-1. The results of numerous studies thus suggest that sodium-dependent bile acid transport can be mediated by both mEH and Ntcp and that additional undefined factors may determine their relative contribution to this process.

In conclusion, the studies reported herein have established that PARP-1 and linker histone H1.2 play a critical role in the transcriptional regulation of EPHX1. The DNA sequences in the binding sites of these regulatory factors serve to further elucidate the mechanism by which they may affect chromatin structure and gene expression by regulating nucleosome positioning. The high frequency of the EPHX1 +2557 polymorphism in the Amish population associated with hypercholanemia suggests that a polymorphism in the mEH gene (EPHX1) contributes, in part, to the etiology of this syndrome and in conjunction with our numerous previous studies [1,3–10,19] strongly supports the critical role of mEH in mediating Na^+^-dependent bile acid transport.
